# A Case of Safe and Effective Use of a Cryoprobe for Simultaneous Biopsy and Removal of a Tracheal Metastasis from Sigmoid Colon Cancer

**DOI:** 10.1159/cro/abzag017

**Published:** 2026-08-17

**Authors:** Reina Asaga-Oniki, Masashi Ishihara, Shigeru Tanzawa, Satoru Ishii, Yoshikane Yamauchi, Shigeki Morita, Yuko Sasajima, Yukinori Sakao, Nobuhiko Seki

**Affiliations:** a Division of Medical Oncology, Department of Internal Medicine, Teikyo University School of Medicine, Tokyo, Japan; b Division of Respiratory Medicine and Allergology, Department of Medicine, Teikyo University School of Medicine, Tokyo, Japan; c Department of Surgery, Teikyo University, Tokyo, Japan; d Department of Pathology, Teikyo University, Tokyo, Japan

**Keywords:** Cryotherapy, Bronchial artery embolization, Tracheal metastasis, Colon cancer

## Abstract

**Introduction:**

Tracheal and endobronchial metastases from extrapulmonary solid tumors are rare. The therapeutic application of cryobiopsy has gradually increased. However, there is still a high risk of bleeding, especially when handling the hypervascular tumor. Our case highlights the novel cryotherapy combined with pre-procedural bronchial artery embolization (BAE) to achieve safe and effective biopsy and removal.

**Case Presentation:**

A 71-year-old man underwent resection for sigmoid colon cancer (pT2N1M0, stage IIIA). Follow-up chest computed tomography (CT), taken due to the cough, revealed a nodular lesion on the lower left wall of the trachea, which gradually enlarged over the subsequent 5 months. Bronchoscopy revealed a broad-based tracheal tumor that accounted for about 50% of the trachea and bled easily upon contact. Dynamic CT also demonstrated that the tumor was fed by a branch of the right bronchial artery. Given the risk of severe bleeding during biopsy and progressive airway obstruction, simultaneous diagnostic biopsy and therapeutic removal, in combination with BAE in advance, were performed. The tumor was removed piecemeal using a cryoprobe, which enabled safe acquisition of unburned tissue without active bleeding and was suitable for broad-based lesions not amenable to high-frequency electrosurgical snares. Histopathological examination confirmed tracheal metastasis from sigmoid colon cancer. In the following year, local recurrence in the trachea and multiple pulmonary metastases were detected, and systemic chemotherapy was initiated.

**Conclusion:**

Cryotherapy may enable safe and effective simultaneous biopsy and removal of broad-based tracheal tumors. Even for hypervascular tumors, combination with pre-procedural BAE may reduce the risk of significant bleeding.

## Introduction

Tracheal and endobronchial metastases from extrapulmonary solid tumors are rare. Marchioni et al. [1] reported that the percentage was only 4%. The most common primary sites include the breast, colorectum, and kidney [1]. Clinical symptoms include cough, hemoptysis, and dyspnea, and progressive disease may result in airway obstruction [2, 3]. Electrocautery, microwave coagulation, and neodymium-doped yttrium aluminum garnet laser therapy have been used to relieve central airway stenosis and obstruction.

Cryobiopsy is a technique in which the tip of a cryoprobe is attached to the tumor, causing the tissue to freeze, adhere to the probe and be removed together with the bronchoscope [4]. Cryobiopsy also allows acquisition of larger specimens than conventional forceps biopsy [5]. Although cryobiopsy has traditionally been used for diagnostic purposes, its application in therapeutic procedures, such as endoscopic debulking, has increased in recent years [4].

Broad-based, hypervascular tracheal metastases present a clinical dilemma: conventional biopsy carries a high bleeding risk, while progressive airway obstruction necessitates timely intervention. We report a case where a cryotherapy in combination with pre-procedural bronchial artery embolization (BAE) enabled safe and effective simultaneous biopsy and removal of a hypervascular tracheal metastasis from sigmoid colon cancer, which suggests that this combination of approaches may reduce the risk of bleeding complications and may result in effective and temporary local control at the same time.

## Case Presentation

A 71-year-old Japanese man underwent high anterior resection and ileocecal resection for sigmoid colon cancer (pT2N1M0, stage IIIA). At the patient’s request, adjuvant chemotherapy was not administered. Two years after the initial surgery, partial resection of liver segment 7 was performed for liver metastasis. Three years later, thoracoscopic right and left upper lobe segmentectomies were performed for bilateral pulmonary metastases.

Two years after lung surgery, the patient developed a cough. Follow-up chest computed tomography (CT) demonstrated a 5.4 mm × 6.8 mm elevated nodule on the lower left wall of the trachea, which increased to 10.6 mm × 8.2 mm in size over the subsequent 5 months (shown in Fig. 1). Bronchoscopy revealed a broad-based tracheal tumor that accounted for about 50% of the trachea and bled easily upon contact (shown in Fig. 2). Dynamic CT also revealed that the tumor was fed by a branch of the right bronchial artery. The differential diagnosis included tracheal metastasis from sigmoid colon cancer and primary tracheal carcinoma, but biopsy at this point without BAE was considered too dangerous because there was a high risk of bleeding. There was also the risk of progressive airway obstruction; therefore, simultaneous diagnostic biopsy and therapeutic removal were planned.

**Fig. 1.**
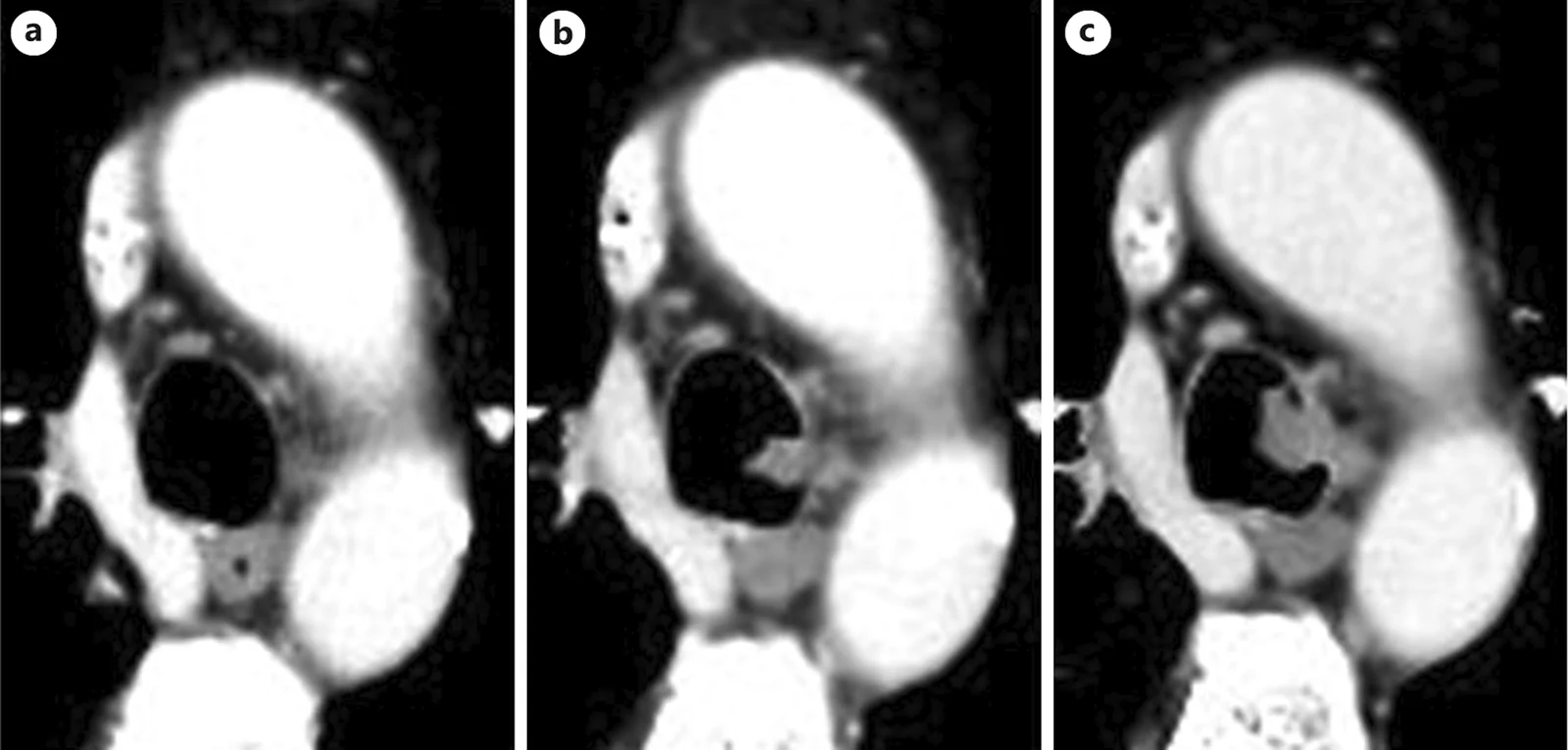
**a** CT performed 4 months before the middle one showed no nodule. **b** CT demonstrated a 5.4 mm × 6.8 mm elevated nodule on the lower left wall of the trachea. **c** The nodule increased to 10.6 mm × 8.2 mm in size 5 months after the middle CT. CT, computed tomography.

**Fig. 2.**
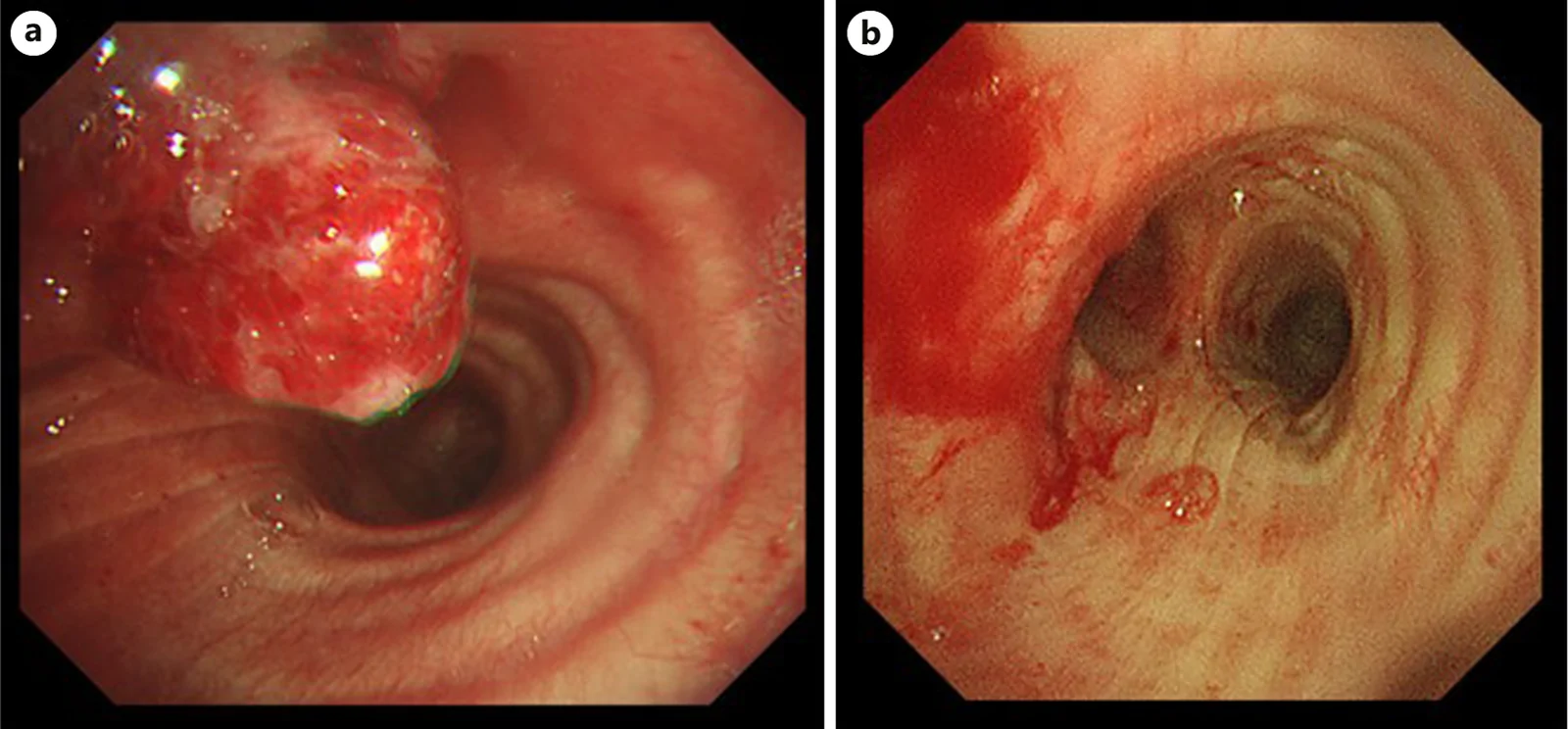
**a** Bronchoscopy revealed a broad-based tracheal tumor and bled easily upon contact. **b** The tumor was removed piecemeal until it was no longer macroscopically visible with no active bleeding.

The patient’s Eastern Cooperative Oncology Group performance status was 1 and he had no comorbidities, no regular medications, and no family history. No stridor was noted on auscultation. Liver enzymes were mildly elevated (AST 74 U/L, ALT 87 U/L), with no other significant laboratory abnormalities. Tumor markers were within normal limits (CEA 1.9 ng/mL, CA 19-9 6.6 U/mL).

To reduce the risk of bleeding during bronchoscopy, BAE was performed prior to the procedure. Post-embolization arteriography confirmed the absence of tumor-feeding vessels. Because the tumor was broad-based, use of a high-frequency electrosurgical snare was considered unsuitable. A cryoprobe was selected to allow safe and simultaneous removal and acquisition of unburned tissue for pathological diagnosis.

Under general anesthesia, a single-use cryoprobe (1.7 mm probe; Erbe Elektromedizin, GmbH, Germany) contacted the tumor with a freezing time of 5–7 s. The tumor was removed piecemeal until it was no longer macroscopically visible, with careful attention to avoid damage to the tracheal wall. No active bleeding occurred during the procedure (shown in Fig. 2). Histopathological examination revealed glandular structures consistent with the previously resected sigmoid colon cancer (shown in Fig. 3), confirming the diagnosis of tracheal metastasis. Although macroscopically complete removal was achieved, the piecemeal nature of the resection (>20 fragments) made it impossible to confirm negative margins histopathologically, which may explain the subsequent local recurrence.

**Fig. 3.**
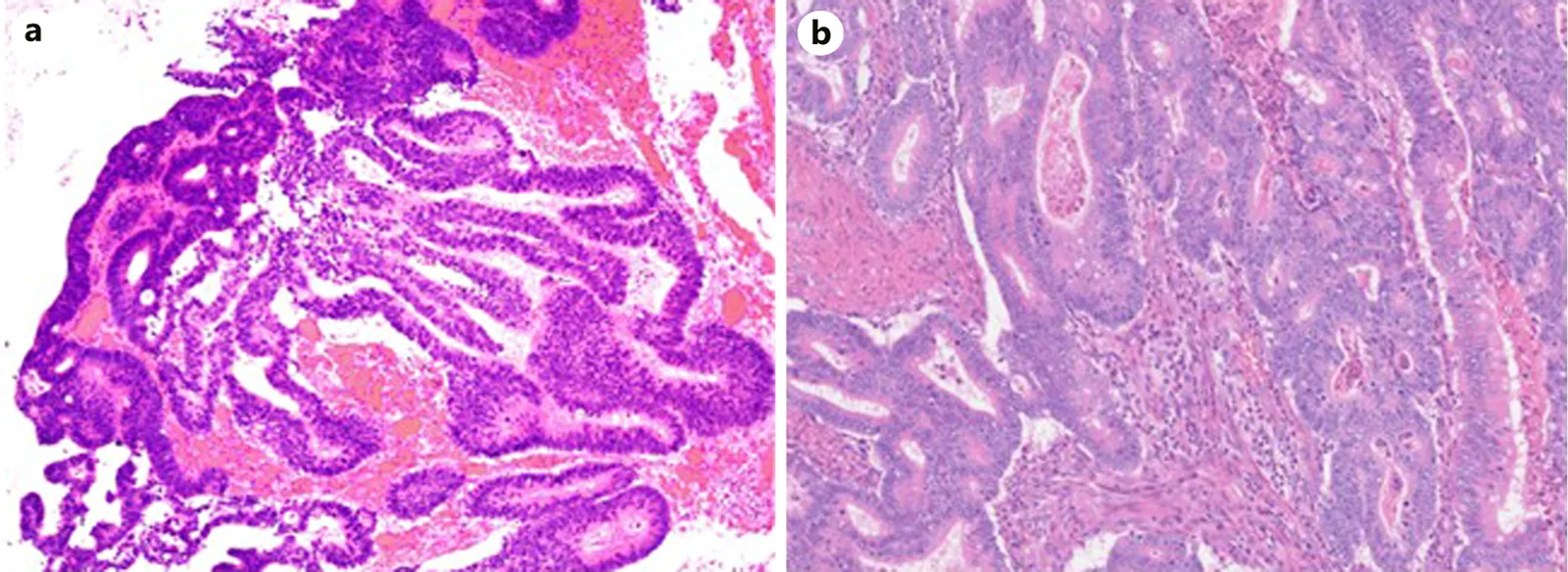
**a** H&E stain (×10 power magnification) shows the glandular structures in the bronchoscopic specimen, which is consistent with the previously resected sigmoid colon cancer. **b** H&E stain (×10 power magnification) shows the glandular structures of sigmoid colon cancer resected 7 years before the bronchoscopy.

Positron emission tomography/CT performed 2 months later showed no evidence of recurrence, and chemotherapy was deferred due to the absence of target lesions. Radiotherapy was avoided to preserve it as a salvage option in case of future local recurrence. However, 6 months after cryotherapy, local recurrence in the trachea and multiple pulmonary metastases were detected. Cryotherapy was performed again, followed by initiation of systemic chemotherapy for the first time during the patient’s treatment course. We completed the CARE checklist, which is provided as an online supplementary file (for all online suppl. material, see https://doi.org/10.1159/cro/abzag017), to ensure comprehensive reporting of our case.

## Discussion

This case demonstrates the safe and effective use of a cryoprobe for simultaneous biopsy and removal of a tracheal metastasis from sigmoid colon cancer. Tracheal and endobronchial metastases from colorectal cancer typically occur within 5 years after diagnosis of the primary tumor and are associated with poor prognosis; Coriat et al. [6] reported that the median survival was 18.9 months. Previous reports have described relief of airway obstruction using surgical resection [7], high-frequency electrosurgical snares [8], or combined modalities including cryoprobes and argon plasma coagulation [9]; however, reports of tumor removal using cryoprobes alone remain limited.

Several bronchoscopic devices are available. However, with the exception of photodynamic therapy for superficial central squamous cell carcinoma, these devices are not curative and are primarily used for symptom relief. Depending on institutional availability, it is important to understand the characteristics of each device and to use them selectively or in combination.

Recently, cryobiopsy has been applied to therapeutic procedures such as endoscopic debulking [4]. Cryobiopsy offers several advantages, including the ability to obtain large, unburned tissue specimens and to treat broad-based tumors not amenable to electrosurgical snares [5, 10]. In our case, the tumor was broad-based; therefore, it was removed using a cryoprobe.

Pathophysiologically, both the bronchial artery and pulmonary artery play important roles in supplying blood to the airways and lungs. Specifically, bronchial arteries are the primary feeding vessels to lung metastases. Cryobiopsy involves rapid freezing of tissue and use of its adhesive properties for extraction, making some degree of local bleeding unavoidable [10]. During endobronchial tumor removal, the incidence of significant bleeding makes it difficult to establish clear procedural boundaries [4]. In addition, there is also a risk of respiratory failure, leading to death.

Pre-procedural BAE may reduce the risk of significant bleeding during cryotherapy [9, 11]. In our case, the tumor was fed by a branch of the right bronchial artery; therefore, BAE in advance may have prevented bleeding complications.

Cryotherapy shows potential for effective and temporary local tumor control. Hamakawa et al. [12] reported 2 cases demonstrating this effect. One involved intratracheal metastasis following postoperative recurrence of lung adenocarcinoma, which was removed using a high-frequency electrosurgical snare and cryoprobes, with no recurrence observed during 5 months of follow-up [12]. The other involved intermediate bronchial metastasis after postoperative recurrence of lung squamous cell carcinoma; the lesion was removed using a high-frequency electrosurgical snare and a cryoprobe, with no recurrence in the intermediate bronchus during 12 months of follow-up [12].

In our case, temporary local control was achieved for 6 months, which allowed deferral of systemic chemotherapy in accordance with the patient’s preference to avoid it. However, we just reported 1 case. There are also several complications associated with BAE such as spinal nerve damage. It would be difficult to conclude the safety and long-term efficacy of cryotherapy in combination with BAE based only on our case. Further cases are needed to establish standardized techniques and elucidate long-term outcomes.

In conclusion, cryotherapy may enable safe and effective simultaneous biopsy and removal of broad-based tracheal tumors. Even for hypervascular tumors, combination with pre-procedural BAE may reduce the risk of significant bleeding during cryotherapy. Additional cases are needed to clarify its long-term safety and efficacy as a temporary local treatment device.

## Statement of Ethics

This study was conducted in accordance with the Declaration of Helsinki. Ethics approval was waived by the Teikyo University Ethics Review Board because this case report was classified as part of routine clinical care and not considered research under institutional ethical guidelines. Written informed consent was obtained from the patient for publication of the details of their medical case and any accompanying images. The writing and presentation of this case report follow the Case Report (CARE) guidelines, and the completed CARE Checklist is provided as online supplementary material.

## Conflict of Interest Statement

The authors have no conflicts of interest to declare.

## Funding Sources

This study was not supported by any sponsor or funder.

## Author Contributions

Reina Asaga, MD: conceptualization, data curation, and writing – original draft; Masashi Ishihara, MD, PhD: conceptualization and writing – review and editing; Shigeru Tanzawa, MD, PhD; Satoru Ishii, MD, PhD; Yoshikane Yamauchi, MD, PhD; Shigeki Morita, MD, PhD: writing – review and editing; Yuko Sasajima, MD, PhD; Yukinori Sakao, MD, PhD; and Nobuhiko Seki, MD, PhD: supervision and writing – review and editing.

## Data Availability

All data generated or analyzed during this study are included in this article and its online supplementary material. Further inquiries can be directed to the corresponding author.
